# The effects of antiretroviral therapy initiation time on HIV reservoir size in Chinese chronically HIV infected patients: a prospective, multi-site cohort study

**DOI:** 10.1186/s12879-019-3847-0

**Published:** 2019-03-14

**Authors:** Ling Luo, Nidan Wang, Yongsong Yue, Yang Han, Wei Lv, Zhengyin Liu, Zhifeng Qiu, Hongzhou Lu, Xiaoping Tang, Tong Zhang, Min Zhao, Yun He, He Shenghua, Min Wang, Yongzhen Li, Shaobiao Huang, Yong Li, Jing Liu, Zhu Tuofu, Jean-Pierre Routy, Taisheng Li

**Affiliations:** 1 Department of Infectious Diseases, Peking Union Medical College Hospital, Chinese Academy of Medical Sciences, No.1 Shuaifuyuan, Wangfujing Street, Beijing, 100730 China; 2 Shanghai Public Health Clinical Center affiliated with Fudan University, Shanghai, China; 3 Guangzhou No.8 People’s Hospital, Guangzhou, China; 4 Beijing You’an Hospital, Capital Medical University, Beijing, China; 5 302 Military Hospital of China, Beijing, China; 6 The Infectious Disease Hospital of Henan Province, Zhengzhou, China; 7 Chengdu Infectious Diseases Hospital, Chengdu, China; 8 The First Hospital of Changsha, Changsha, China; 9 The Center for Disease Prevention and Control of Guangxi province, Nanning, China; 10 Nanning No.4 People’s Hospital, Nanning, China; 11 The Longtan Hospital, Liuzhou, China; 12 The hospital affiliated with the Chinese Medical University, Hangzhou, China; 13 Department of Laboratory Medicine, University of Washington, Seattle, USA; 14 Research Institute of the McGill University Health Centre, Chronic Viral Illness Service, and Division of Hematology, McGill University Health Centre, Montreal, Québec Canada

**Keywords:** HIV-1, DNA reservoir, Antiretroviral therapy, Initiation time

## Abstract

**Background:**

The effect of ART initiation time on HIV-1 DNA reservoir in chronically infected individuals is not well understood. Determining the potential influencing factors associated with a low HIV-1 DNA level in chronic infection is an important step toward drug-free control.

**Methods:**

A prospective study included 444 chronically HIV-infected adults was performed. Participants were divided into two groups: early initiation group (EIG) or delayed initiation group (DIG) based on their baseline CD4 count; 350 to 500 and < 350 cells/mm^3^, respectively. Total HIV-1 DNA was measured by quantitative PCR. Using the Mann-Whitney U test, the HIV-1 DNA level at week 48 was compared between the two groups. The influencing factors of the HIV-1 DNA and factors associated with achieving a low HIV-1 level at week 48 were analyzed.

**Results:**

The HIV-1 DNA at week 48 in EIG was significantly lower than in DIG [2.12 (1.80–2.51) vs 2.58 (2.21–2.87) log_10_ copies/10^6^peripheral blood mononuclear cells (PBMCs); *p* = 0.001]. Early ART initiation was positively associated with lower HIV-1 DNA at week 48 (*p* = 0.025). Similarly, baseline HIV-1DNA (*p* = 0.001) was positively associated with HIV-1DNA at week 48 and baseline CD4/CD8 ratio (*p* = 0.001) was inversely associated with HIV-1DNA at week 48. Early ART initiation (*p* = 0.003) and baseline HIV-1 DNA level (*p* < 0.001) were positively associated with achieving HIV-1 DNA < 100 copies/10^6^ PBMCs at week 48.

**Conclusion:**

Early ART initiation is positively associated with a smaller size of viral reservoir and a higher possibility of achieving a low HIV-1DNA level at week 48 in Chinese chronically HIV-1 infected adult.

**Trial registration:**

NCT01844297; Registered 1 May, 2013.

## Background

With more than 36 million persons living with HIV (PLHIV) globally [1], the number of PLHIV on antiretroviral treatment (ART) and related healthcare costs are gradually rising. Consequently, the international HIV cure initiative seeks a therapy-free solution [2, 3]. A major obstacle to HIV remission is the persistence of the virus as integrated HIV-1 DNA in infected memory CD4+ T cells even after long-term cART [2, 4]. Working on eradication or permanent control strategies requires HIV reservoir quantification and monitoring that is more informative than routine plasma HIV-1RNA testing.

The size of the HIV-1 DNA reservoir is predictive of clinical outcomes and disease progression, independent of CD4 cell count and HIV-1 RNA load [5]. Very low HIV-1 DNA are observed in 2 particular populations of HIV-infected individuals: the elite HIV controllers (who control spontaneously viral replication) [6] and the posttreatment controllers (who initiate ART during early infection and are subsequently able to control viral replication for several years after ART interruption) [7]. Thus, reducing the HIV-1 DNA reservoir as much as possible can be an interesting aim, as it could be a criterion for ART reduction or interruption [8]. HIV-1 DNA level is also considered to be associated with non-AIDS related morbidities [9] and mortalities in virally suppressed patient [10].

Many approaches are used to estimate the size of HIV-1 reservoirs including quick PCR techniques to laborious viral outgrowth assays [11]. Among them, total HIV-1 DNA assay is a reproducible and standardized marker [12–14]. Furthermore, total HIV-1 DNA quantification is better suited large clinical trials compared to the use of labor-intensive viral outgrowth assay [11]. Although still unavailable worldwide as a standardized assay, total HIV-1 DNA quantification is becoming cheaper and more accessible. It has been applied to cases of special interest including early therapy initiation in adults [4, 11, 15, 16] and infants [16–18], controlled treatment interruption [5], post-treatment controllers [19] and intensification of regimens [20, 21].

Treatment as early as possible during primary HIV-1 infection restricts the size of HIV reservoirs, ensuring optimal immune restoration and inhibiting T-cell activation [4, 21, 22]. In comparison, HIV reservoir is more stable in patients with chronic HIV infection [23], but unfortunately, most patients infected with HIV are diagnosed at this stage. The effect of ART initiation time on the reservoir size in chronic infection is not well understood. Several studies describe HIV-1 DNA levels in treated patients but are often restricted by single time-point sampling and number of patients [8, 23–27]. Furthermore, recent related studies include Caucasians primarily while data on Asians are insufficient [23, 25, 26, 28–30]. The difference in the dominance in HIV-1 subtype between Chinese patients and Caucasian patients may lead to the difference in the amount of HIV-1 DNA change after cART between these two different ethnic groups. A research conducted in Chinese HIV-infected patients shows that the subtype of HIV-1 influenced the amount of HIV-1 DNA change after cART. In this study, after 18 months of cART, total HIV-1 DNA decreased more pronouncedly in patients infected by CRF01_AE than in those infected by subtype B and CRF07_BC [31]. As we know, CRF01_AE is a dominant strain among native HIV-infected individuals in China [32], whereas subtype B dominates the HIV-1 epidemic in North America and in Western and Central Europe [33]. Therefore, we undertook a prospective multi-site cohort study of HIV-positive Chinese adults with chronic infection to explore the effect of ART initiation time on the total HIV-1 DNA after ART. We also explore the potential influencing factors associated with a low HIV-1 DNA level in chronic infection.

## Methods

### Subjects

The China AIDS Clinical Trial 1215 study (CACT1215) is a prospective, multicenter cohort study designed to compare the efficacy of cART with different initiation time and assess the safety of ART regimens. The CACT1215 study was conducted in clinical trial units located in 9 Chinese cities: Beijing, Shanghai, Guangzhou, Chengdu, Changsha, Nanning, Liuzhou, Zhengzhou and Shenyang. The patients therefore represent a broad cross-section of the overall population of HIV infected patients in China. ART-naïve individuals with documented HIV-1 infection, who were between 18 and 65 years of age, and who had CD4 counts ≤500 cells/mm^3^ were eligible for the study. After baseline assessment, participants were treated 300 mg of lamivudine, 600 mg of tenofovir and 600 mg of efavirenz, daily, within two weeks of enrollment. On the basis of baseline CD4 count, patients were divided into 2 groups: HIV-positive individuals who had a baseline CD4 count of either 350–500 cells/mm^3^ (early initiation group, EIG) or baseline CD4 count < 350 cells/mm^3^ (delayed initiation group, DIG). As part of this large cohort study, the HIV-1DNA levels of the participants were measured prior to ART initiation, after 24 weeks and 48 weeks of treatment. From a total of 500 patients enrolled in this study, 444 patients had completed all the HIV-1 DNA measurements and were included in the present analysis.

The study protocol was approved by an independent ethics committee and the institutional review board of PUMCH (Peking Union Medical College Hospital). The trial was carried out in accordance with the principles of Good Clinical Practice and the Declaration of Helsinki. Written informed consent was obtained from all the participants. This study was registered with ClinicalTrials.gov, number NCT01844297.

### HIV-1 DNA quantification

Total HIV-1 DNA was extracted from 200 μL peripheral blood using Qiagen QIAsymphony DNA Mini Kits (QIAGEN, Valencia, CA). The extraction of HIV-1 DNA and PCR for HIV-1 DNA were made using frozen samples. HIV-1 DNA in the peripheral blood (mainly white blood cells, WBCs) was amplified and quantified for LTR gene using a fluorescence-based, real-time SUPBIO HIV Quantitative Detection Kit (SUPBIO, Guangzhou, China). The reaction system as follows: reaction mixture 44.2 μL, enzyme 0.8 μL, DNA 5 μL. The housekeeping gene were amplified at the same time to quantify the cell amount. HIV-1 DNA were measured in duplicate and the quantification range of this assay was 20–5 × 10^6^ copies/10^6^ WBCs. The amount of HIV-1 DNA per 10^6^ PBMCs was calculated.

### CD4+ cells count, CD8+ cells count and HIV-1 RNA determination

CD4+ T lymphocytes and CD8+ T lymphocytes were determined by flow cytometry (FACS Canto, BD Biosciences, NJ, USA) using commercially available monoclonal antibodies and plasma HIV-1 RNA load was measured using the COBAS Ampliprep/TaqMan 48 real-time RT-PCR Test (Roche, CA, USA) according to the manufacturer’s instructions. The detection range was from 40 to 1,000,000 copies/mL. All participants were tested for CD4+ and CD8+ cell counts and HIV-1RNA at baseline and at time of all visit.

### Statistical analysis

Demographic and baseline clinical characteristics were summarized for each treatment group using the median and the interquartile range (IQR) for continuous variables and the frequency and the percentage for categorical variables. We used the Mann-Whitney U test to compare the distribution of HIV-1 DNA (log10 /106 PBMCs) between the two groups at week 24 and at week 48. We used the Mann-Whitney U test to analyze the distribution of changes in the HIV-1 DNA from baseline to week 24 and week 48 between the two groups. Mixed-effects regression modeling was performed to assess the impact of ART timing on HIV-1 DNA at week 48; this was analyzed on a logarithmic scale (log_10_). Both univariate and multivariate models were used to determine the relative associations between early versus delayed ART, age, sex, baseline CD4+ cell count, baseline CD4/CD8 ratio, baseline HIV-1 RNA, baseline HIV-1 DNA and HIV-1 DNA at week 48. Univariate and multivariate logistic regression were used to identify predictors of a low HIV-1 DNA level at week 48. Statistical analysis was performed using SPSS 22.0 (IBM Corporation, Armonk, New York, USA) and Stata/SE 13.0 software (StataCorp. College Station/USA). A *p* value of < 0.05 was considered significant.

## Results

### Baseline participant characteristics

Table 1 summarized the baseline characteristics of the 444 Chinese adult ART-naïve patients whose data were included in the study. There were 265 participants with CD4 count < 350 cells/mm^3^ in DIG and 179 subjects with CD4 count between 350 and 500 cells/mm^3^ in EIG. The EIG had higher proportion of HIV-1 AE subtype than DIG (47.2% vs. 33.5%, *p* = 0.004). The median baseline CD4 count in EIG was higher than DIG (401 vs. 239 cells/mm^3^, *p* < 0.001). The baseline CD4/CD8 ratio was also higher in EIG (0.39 vs.0.25, *p* = 0.001). The median baseline HIV-1 DNA was also lower in the EIG than DIG (2.73 vs. 3.00 log_10_ copies/10^6^ PBMCs, *p* < 0.001).

**Table 1:** Selected Demographic and Baseline Characteristics

	DIG(*N* = 265)	EIG(*N* = 179)	*P* value
Age: years (IQR)	33(27–42)	32(27–40)	0.276
Male: no. (%)	199(75.1)	125 (69.8)	0.232
Ethnicity:no. (%)			
Han	195(73.6)	123(68.1)	0.284
Other	70 (26.4)	56 (31.9)	
Mode of HIV acquisition: no. (%)			
MSM	92 (34.7%)	63 (35.2%)	0.920
Heterosexual	142 (53.6%)	98 (54.7%)	0.846
Bisexual	6 (2.3%)	6 (3.4%)	0.557
Blood products	2 (0.4%)	1 (0.6%)	1.000
Other/undefined	14 (9.3%)	11 (5.5%)	0.682
HIV-1 subtype			
AE	125 (47.2%)	60 (33.5%)	0.004
B/C/BC	69 (26.0%)	63 (35.2%)	0.044
unknown	71 (26.8%)	56 (31.3%)	0.336
CD4 count: (cells/mm^3^)	239 (161–292)	401 (376–451)	< 0.001
CD4/CD8 ratio:	0.25 (0.19–0.39)	0.39 (0.24–0.45)	0.001
HIV-1 RNA: (log_10_ copies/mL)	4.83 (4.43–5.14)	4.66 (4.39–5.05)	0.641
HIV-1 DNA: (log_10_ copies /10^6^PBMCs)	3.00 (2.65–3.39)	2.73 (2.40–3.03)	< 0.001
HBsAg (+): no. (%)	27 (10.2%)	18 (10.1%)	1.000
HCV-Ab (+): no. (%)	9 (3.4%)	4 (2.2%)	0.579

Data are number (%) or median (IQR). MSM: men who have sex with men; DIG: delayed initiation group; EIG: early initiation group; HIV: human immunodeficiency virus; IQR: interquartile range; PBMC: peripheral mononuclear cell; HBsAg: Hepatitis B surface antigen; HCV: Hepatitis C virus; Ab: antibody

### HIV-1 DNA dynamics

**Fig. 1:**
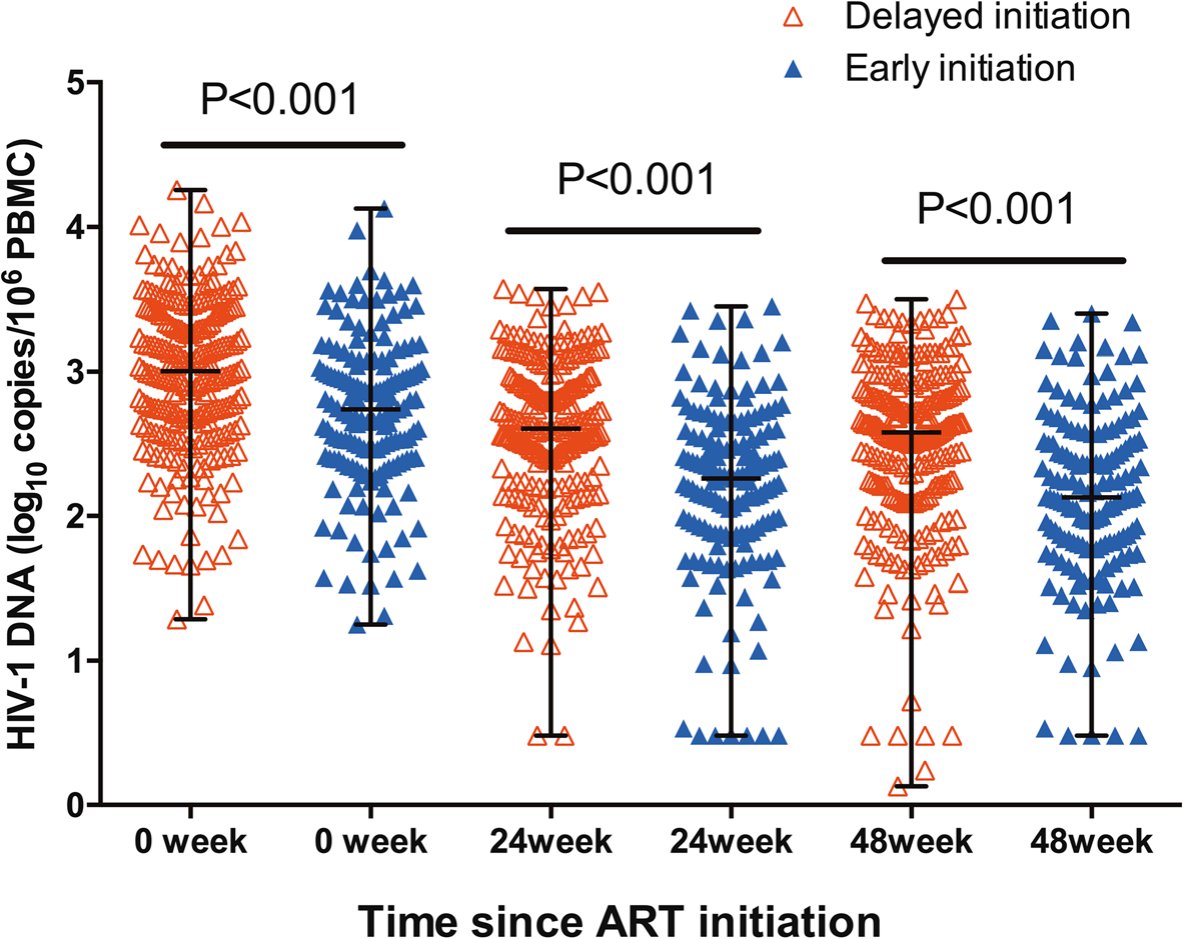
Comparation of HIV-1 DNA at different time points between EIG and DIG

At week 48, the median HIV-1 DNA in the EIG was significantly lower than that in the DIG [2.12 (1.80–2.51) vs. 2.58 (2.21–2.87) log_10_ copies/10^6^ PBMCs, *p* < 0.001; Fig. 1]. At week 24, the median HIV-1 DNA in the EIG was also significantly lower than that in the DIG [2.26 (1.89–2.59) vs. 2.61(2.25–2.93) log_10_ copies/10^6^ PBMCs, *p* < 0.001; Fig. 1].

The largest decrease in HIV-1 DNA occurred during the first 24 weeks in both groups: at week 24, the median change from baseline in HIV-1 DNA was − 0.46 (− 0.69 to − 0.22) log_10_ copies/10^6^ PBMCs in the EIG and − 0.44 (− 0.66 to − 0.20) log_10_ copies/10^6^ PBMCs in the DIG. At week 48, the median change from baseline in HIV-1 DNA was − 0.48 (− 0.81 to − 0.25) log_10_ copies/10^6^ PBMCs in the EIG and − 0.48 (− 0.72 to − 0.25) log_10_ copies/10^6^ PBMCs in the DIG.

After 24 weeks of cART, 97.8% (175/179) of the participants achieved HIV-1 RNA < 400 copies/ml after 48 weeks of ART in the EIG and 93.2% (247/265) of the participants achieved HIV-1 RNA < 400 copies/ml after 48 weeks of ART in the DIG. After 48 weeks of cART, 98.9% (177/179) of the participants achieved HIV-1 RNA < 400 copies/ml in the EIG and 95.1% (252/265) achieved HIV-1 RNA < 400 copies/ml in the DIG (*p* = 0.033).

### Effects of ART initiation time on the HIV-1 DNA at week 48

We examined how the HIV-1 DNA at week 48 was influenced by ART initiation time (EIG vs. DIG), age, sex, the baseline CD4 count, the baseline CD4/CD8 ratio, the baseline HIV-1 RNA and the baseline HIV-1 DNA. Multivariate Mixed-effects modeling showed lower HIV-1 DNA level at week 48 was predicted by earlier ART initiation time (*p* = 0.025), lower baseline HIV-1 DNA level (*p* = 0.001), and higher baseline CD4/CD8 ratio (*p* = 0.001); Table 2.

**Table 2:** Predictors of HIV-1 DNA Level at Week 48

	HIV-1 DNA at week 48 (Log_10_ copies/10^6^PBMCs)		
Predictor	Estimate	95% CI	*P* Value
Univariate Model			
Early ART Initiation	−0.309	− 0.408 to − 0.210	0.001
Age (years)	+ 0.007	+ 0.002 to + 0.013	0.01
Baseline HIV-1 RNA (log_10_ copies/mL)	+ 0.345	+ 0.268 to + 0.423	0.001
Baseline HIV-1 DNA (log_10_ copies /10^6^PBMCs)	+ 0.696	+ 0.633 to + 0.759	0.001
Baseline CD4/CD8 ratio	−0.643	− 0.955 to − 0.332	0.001
Model with Early ART initiation, age, baseline HIV-1 RNA, baseline HIV-1 DNA and baseline CD4/CD8 ratio			
Early ART initiation	−0.092	−0.173 to − 0.011	0.025
Age(years)	+ 0.002	−0.002 to + 0.006	0.339
Baseline HIV-1 RNA (log_10_ copies/mL)	+ 0.026	−0.061 to + 0.114	0.553
Baseline HIV-1DNA (log_10_ copies /10^6^PBMCs)	+ 0.631	+ 0.545 to + 0.717	0.001
Baseline CD4/CD8 ratio	−0.309	−0.448 to − 0.169	0.001

### Factors associated with a low HIV-1 DNA level at week 48

Because there is no standard to define a low HIV-1 DNA level, two cut-offs were considered: HIV-1DNA level below the limit of detection (20 copies/10^6^PBMCs) and lower than 100 copies/10^6^ PBMCs, which was used in Slim Fourati et al’ study [8]. After 48 weeks of cART, the percentage of subjects who achieved HIV-1 DNA below the limit of detection was also significantly higher in EIG than DIG (7.3% vs. 3.0%, *p* = 0.039) and the percentage of subjects who achieved HIV-1 DNA lower than 100 copies/10^6^PBMCs was also significantly higher in EIG than DIG (37.4% vs. 17.7%, *p* < 0.001).

To assess whether clinical, immunological or virological parameters might be related to obtaining a low HIV-1 DNA level at week 48, univariate and multivariate logistic models were used. We examined how the percentage of participants who achieved a low HIV-1 DNA level at week 48 was influenced by ART initiation timing, age, sex, HBV infection, HCV infection, the baseline CD4 count, the baseline CD4/CD8 ratio, the baseline HIV-1 RNA and the baseline HIV-1 DNA. When considering the cut-off 20 copies/10^6^PBMCs, a low HIV-1DNA level at week 48 was associated with the baseline HIV-1 DNA level (*p* < 0.001); Table 3. When considering the cut-off lower than 100 copies/10^6^ PBMCs, a low HIV-1DNA at week 48 was associated with early ART initiation (*p* = 0.003) and the baseline HIV-1 DNA level (*p* < 0.001); Table 3.

**Table 3:** Multivariate Logistic Regression Analysis of Factors Associated with A Low HIV-1 DNA Level at Week 48

	OR	95% CI	*P* value
Variable associated with a low HIV-1 DNA level (< 20 copies/10^6^ PBMCs)			
Baseline HIV-1 DNA (log_10_ copies /10^6^PBMCs)	0.123	0.052–0.292	< 0.001
Variable associated with a low HIV-1 DNA level (< 100 copies/10^6^PBMCs)			
EIG	2.319	1.348–4.127	0.003
Baseline HIV-1 DNA (log_10_ copies /10^6^PBMCs)	0.039	0.019–0.081	< 0.001

*Abbreviations*: *EIG* early initiation group

## Discussion

In this study, we demonstrate that chronically infected adults who start ART at a CD4 count between 350 and 500 cells/mm^3^ had a lower HIV-1 DNA reservoir size and a higher possibility of achieving a low HIV-1DNA level, compared to those patients who started ART at a CD4 count < 350 cells/mm^3^. To our knowledge, this is the first multicenter prospective cohort study in China [26] and the prospective cohort study with the largest participant number all over the world [8, 21, 25, 34, 35], to comprehensively assess the effect of different ART initiation time on HIV-1 DNA reservoir size after cART in chronically-infected patients.

Cellular reservoirs of latent, genomically-integrated HIV are established quickly after infection [8]. HIV-1-infected individuals who achieve virologic suppression with cART nonetheless retain long-lived cellular HIV reservoirs. For individuals with a peak viral load below 5 log_10_ HIV-1 RNA copies/mL, a past episode of virologic failure is associated with an increased risk of having high HIV-1 DNA level [5]. A low total HIV-1 DNA in PBMCs is independently predictive of a longer time to loss of viral control following treatment interruption [8]. Given the correlation between HIV-1 DNA levels and clinical outcomes [5] and given that replication incompetent virus can contribute to immune activation [36–38], controlling this latent HIV-1 reservoir is an important goal of future HIV treatment strategies.

Our results showed a decline in the size of HIV-1 reservoirs was reported at weeks 24 and 48; however, this observed decrease was less than that previously reported in individuals with primary infection [39]. The decrease in total HIV-1 DNA reservoirs did not differ significantly between the EIG and the DIG after ART initiation.

Defining the interplay between ART initiation time and size of viral reservoir is critical. Our multivariate modeling demonstrates that ART initiation time, the baseline HIV-1 DNA levels, and the baseline CD4/CD8 ratio are associated with the HIV-1 DNA level following ART. Our results demonstrate a significant correlation between early ART initiation and a lower level of HIV-1 DNA at follow-up time after ART, suggesting lower viral reservoirs levels would be achieved by starting ART earlier in chronically-infected patients. In our study, low levels of pretreatment HIV-1 DNA were correlated with low levels of HIV-1 DNA at follow-up time after cART, which was consistent with the previous study [21, 23, 40]. Our study also showed that a higher pre-ART CD4/CD8 ratio was associated with a low HIV-1 reservoir after cART. Chun et al. first revealed an inverse correlation between the CD4/CD8 ratio and CD4+ T cells carrying HIV-1 DNA in infected patients receiving cART [41]. Later, Boulassel et al. confirmed this result [42]. One possible explanation is that a low baseline CD4/CD8 ratio indicates a high extent of immune activation and enhanced homeostatic proliferation of HIV-1-infected CD4+ lymphocyte, resulting in the high level of the persistence of HIV-1DNA reservoirs [43, 44]. The amount of HIV-1 DNA is influenced by the CD4/CD8 ratio [45]. Early ART initiation is often associated with a higher probability of normalization of the CD4/CD8 ratio after cART [46]. The mechanisms underlying the association of early ART initiation and the level of HIV-1 DNA after cART could be that early ART initiation can lead to a higher percentage of normalization of the CD4/CD8 ratio after cART, which can further influenced the level of HIV-1 DNA after cART.

Low levels of HIV-1 DNA are predictive of better clinical outcomes in infected individuals [5]. Bring HIV-1 DNA to extremely low levels may permit treatment simplification [34] and influence the occurrence of viral rebound upon discontinuation of therapy [47]. Those individuals with very low levels of HIV-1 DNA might be the ideal population to enroll in future cure trials involving reduced or interrupted ART [8].

Regardless of the cut-off used, the percentage of subjects achieved a low HIV-1 DNA level at week 48 was significantly higher in the EIG than that in the DIG. When considering the cut-off of 100 copies/10^6^ PBMCs, our multivariate modeling suggests that early ART initiation and the low baseline HIV-1 DNA are associated with achieving a low HIV-1 DNA level at week 48. Considering that a low HIV-1 DNA level is meaningful for functional cure, our study may contribute to the selection and the monitoring of patients on ART who will be selected to participate in eradication studies [29].

Our study has several limitations. First, as with any observational study, even after adjusting for known possible risk factors, residual confounding may occur because of unmeasured risk factors that may be associated with early ART initiation and lower level of HIV-1 DNA. Only a large, well-designed randomized trial can balance such unmeasured factors. Second, follow-up was limited to 48 weeks. The duration of cART in our study was not sufficient to fully understand the long-term effects of the ART initiation time on the HIV-1 DNA reservoir. However, the reservoir stayed stable after 6 months of cART. Longer prospective studies are needed, therefore, to assess the long-term effect of different ART initiation strategies on reducing the size of the HIV reservoir.

## Conclusions

In conclusion, a lower HIV-1 DNA following cART is found to be associated with early ART initiation, with a lower baseline HIV-1 DNA and with higher baseline CD4/CD8 ratio in chronically infected adults. Factors associated with HIV-1 DNA lower than 100 copies/10^6^PBMCs at week 48 include ART initiation time and the baseline HIV-1 DNA level. This study provides more information on HIV-1 DNA reservoir related factors in chronic infection. Considering a low HIV-1 DNA level is a meaning factor for functional cure, our study may contribute to the selection and the monitoring of patient who could be selected to participate in the viral eradication studies.

## Abbreviations

Ab: Antibody
ART: Antiretroviral therapy
CACT1215: China AIDS Clinical Trial 1215 study
DIG: Delayed initiation group
EIG: Early initiation group
HBsAg: Hepatitis B surface antigen
HCV: Hepatitis C virus
HIV: Human immunodeficiency virus
IQR: Interquartile range
PBMC: Peripheral blood mononuclear cells
PLHIV: Persons living with HIV
PUMCH: Peking Union Medical College Hospital
